# Supplementary material for: Genome Size in the Arenaria ciliata Species Complex (Caryophyllaceae), with Special Focus on Northern Europe and the Arctic
Source: Plants (Basel). 2024 Feb 26;13(5):635. doi: 10.3390/plants13050635 (PMC10934573; doi:10.3390/plants13050635)
Supplement: Supplementary file 1 [file plants-13-00635-s001.zip › plants-2842517-supplementary.pdf]

Supplementary Materials

Genome Size in the *Arenaria ciliata* Species Complex (Caryophyllaceae), with Special Focus on Northern Europe and the Arctic

Kozlowski et al.

Table S1. Characterization of all collected taxa and samples from the *Arenaria ciliata* species complex. with the corresponding genome sizes (2c values in pg of DNA and the nuclei number per analyzed sample).

Taxon	Coll_date	Lat	Lon	Region	Country	Elev	2c	Nuclei
Arenaria pseudofrigida	20.07.2023	72.50517	-23.95197	Traill Island	Greenland	100	1.61	203
Arenaria pseudofrigida	20.07.2023	72.50517	-23.95248	Traill Island	Greenland	100	1.58	178
Arenaria pseudofrigida	20.07.2023	72.50514	-23.9525	Traill Island	Greenland	100	1.58	289
Arenaria pseudofrigida	20.07.2023	72.50523	-23.9524	Traill Island	Greenland	100	1.61	186
Arenaria pseudofrigida	20.07.2023	72.50525	-23.95214	Traill Island	Greenland	100	1.63	368
Arenaria pseudofrigida	20.07.2023	72.50111	-24.00326	Traill Island	Greenland	10	1.58	188
Arenaria pseudofrigida	20.07.2023	72.50117	-24.00292	Traill Island	Greenland	10	1.63	121
Arenaria pseudofrigida	20.07.2023	72.5004	-24.00373	Traill Island	Greenland	10	1.63	307
Arenaria pseudofrigida	20.07.2023	72.50101	-24.00321	Traill Island	Greenland	10	1.66	162
Arenaria pseudofrigida	20.07.2023	72.50111	-24.00335	Traill Island	Greenland	10	1.58	116
Arenaria pseudofrigida	20.07.2023	72.50113	-24.00331	Traill Island	Greenland	10	1.54	352
Arenaria pseudofrigida	25.07.2023	75.143	-19.734184	Hochstetter	Greenland	30	1.84	198
Arenaria pseudofrigida	25.07.2023	75.143	-19.734184	Hochstetter	Greenland	30	1.77	757
Arenaria pseudofrigida	25.07.2023	75.143	-19.734184	Hochstetter	Greenland	30	1.78	312
Arenaria pseudofrigida	25.07.2023	75.143	-19.734184	Hochstetter	Greenland	30	1.67	148
Arenaria pseudofrigida	25.07.2023	75.143	-19.734184	Hochstetter	Greenland	30	1.69	128
Arenaria pseudofrigida	25.07.2023	75.143	-19.734184	Hochstetter	Greenland	30	1.69	235
Arenaria pseudofrigida	25.07.2023	75.143	-19.734184	Hochstetter	Greenland	30	1.61	410
Arenaria pseudofrigida	25.07.2023	75.143	-19.734184	Hochstetter	Greenland	30	1.63	388
Arenaria pseudofrigida	25.07.2023	75.143	-19.734184	Hochstetter	Greenland	30	1.69	170
Arenaria pseudofrigida	25.07.2023	75.143	-19.734184	Hochstetter	Greenland	30	1.71	989
Arenaria norvegica	24.06.2023	64.690833	-21.453611	Borgarnes	Iceland	60	2.89	728
Arenaria norvegica	24.06.2023	64.690833	-21.453611	Borgarnes	Iceland	60	2.81	269
Arenaria norvegica	24.06.2023	64.690833	-21.453611	Borgarnes	Iceland	60	2.80	203

Arenaria norvegica	24.06.2023	64.690833	-21.453611	Borgarnes	Iceland	60	2.77	192
Arenaria norvegica	24.06.2023	65.5775	-22.167778	Reykholahreppur	Iceland	50	2.68	141
Arenaria norvegica	24.06.2023	65.5775	-22.167778	Reykholahreppur	Iceland	50	2.81	417
Arenaria norvegica	24.06.2023	65.5775	-22.167778	Reykholahreppur	Iceland	50	2.80	228
Arenaria norvegica	24.06.2023	65.5775	-22.167778	Reykholahreppur	Iceland	50	2.73	119
Arenaria norvegica	24.06.2023	65.5775	-22.167778	Reykholahreppur	Iceland	50	2.78	215
Arenaria norvegica	25.06.2023	65.559722	-24.003056	Latrabjarg	Iceland	30	2.76	310
Arenaria norvegica	25.06.2023	65.559722	-24.003056	Latrabjarg	Iceland	30	2.86	153
Arenaria norvegica	25.06.2023	65.559722	-24.003056	Latrabjarg	Iceland	30	2,78	179
Arenaria norvegica	25.06.2023	65.559722	-24.003056	Latrabjarg	Iceland	30	2,78	325
Arenaria norvegica	25.06.2023	65.559722	-24.003056	Latrabjarg	Iceland	30	2,81	178
Arenaria norvegica	25.06.2023	65.559722	-24.003056	Latrabjarg	Iceland	30	2,80	117
Arenaria norvegica	25.06.2023	65.5275	-24.4375	Latrabjarg	Iceland	90	2,83	196
Arenaria norvegica	25.06.2023	65.5275	-24.4375	Latrabjarg	Iceland	90	2,79	172
Arenaria norvegica	25.06.2023	65.5275	-24.4375	Latrabjarg	Iceland	90	2,79	169
Arenaria norvegica	25.06.2023	65.5275	-24.4375	Latrabjarg	Iceland	90	2,75	198
Arenaria norvegica	25.06.2023	65.5275	-24.4375	Latrabjarg	Iceland	90	2,83	248
Arenaria norvegica	21.06.2010	58.124883	-4.9229	Scotland	UK	300	2,87	181
Arenaria gothica	06.07.2017	58.004167	18.563056	Gotland	Sweden	68	4,41	162
Arenaria gothica	07.07.2017	57.854167	18.865833	Gotland	Sweden	9	4,33	239
Arenaria gothica	06.07.2017	58.004167	18.563056	Gotland	Sweden	68	3,94	212
Arenaria gothica	07.07.2017	57.854167	18.865833	Gotland	Sweden	9	3.9	291
Arenaria multicaulis	18.08.2023	46.403889	7.613611	Daubensee, VS	Switzerland	2220	1.57	160
Arenaria multicaulis	18.08.2023	46.404444	7.615833	Daubensee, VS	Switzerland	2220	1.45	139
Arenaria multicaulis	18.08.2023	46.406111	7.619722	Daubensee, VS	Switzerland	2230	1.58	273
Arenaria multicaulis	18.08.2023	46.419444	7.625556	Daubensee, VS	Switzerland	2260	1.43	146
Arenaria multicaulis	18.08.2023	46.398056	7.576389	Daubensee, VS	Switzerland	2330	1.49	281
Arenaria c. subsp. *bernensis*	06.09.2023	46.5143	7.13803	Pointe de Paray, VD	Switzerland	2319	6.86	570
Arenaria c. subsp. *bernensis*	06.09.2023	46.51414	7.13755	Pointe de Paray, VD	Switzerland	2343	7.02	264
Arenaria c. subsp. *bernensis*	06.09.2023	46.51363	7.1375	Pointe de Paray, VD	Switzerland	2361	6.77	788
Arenaria c. subsp. *bernensis*	14.08.2023	46.43267	6.9766	Rochers de Naye, VD	Switzerland	2022	6.65	368

Arenaria c. subsp. *bernensis*	14.08.2023	46.43264	6.97657	Rochers de Naye, VD	Switzerland	2020	6.51	374
Arenaria c. subsp. *bernensis*	14.08.2023	46.43268	6.97656	Rochers de Naye, VD	Switzerland	2020	6.53	106
Arenaria c. subsp. *bernensis*	14.08.2023	46.43311	6.97718	Rochers de Naye, VD	Switzerland	2020	6.74	118
Arenaria c. subsp. *bernensis*	14.08.2023	46.4331	6.97717	Rochers de Naye, VD	Switzerland	2020	6.68	171
Arenaria c. subsp. *bernensis*	14.08.2023	46.43309	6.97719	Rochers de Naye, VD	Switzerland	2020	6.71	139
Arenaria c. subsp. *bernensis*	16.08.2023	46.528	7.20379	Dent de Combettes, VD	Switzerland	2079	7.02	119
Arenaria c. subsp. *bernensis*	16.08.2023	46.52812	7.20352	Dent de Combettes, VD	Switzerland	2012	6.96	167
Arenaria ciliata s.str.	16.08.2023	46.52403	7.19905	Rochers des Rayes, VD	Switzerland	1940	1.57	215
Arenaria ciliata s.str.	16.08.2023	46.52411	7.19919	Rochers des Rayes, VD	Switzerland	1955	1.67	227
Arenaria ciliata s.str.	16.08.2023	46.52415	7.19921	Rochers des Rayes, VD	Switzerland	1955	1.69	254
Arenaria ciliata s.str.	16.08.2023	46.52812	7.20356	Dent de Combettes, VD	Switzerland	2012	1.60	531

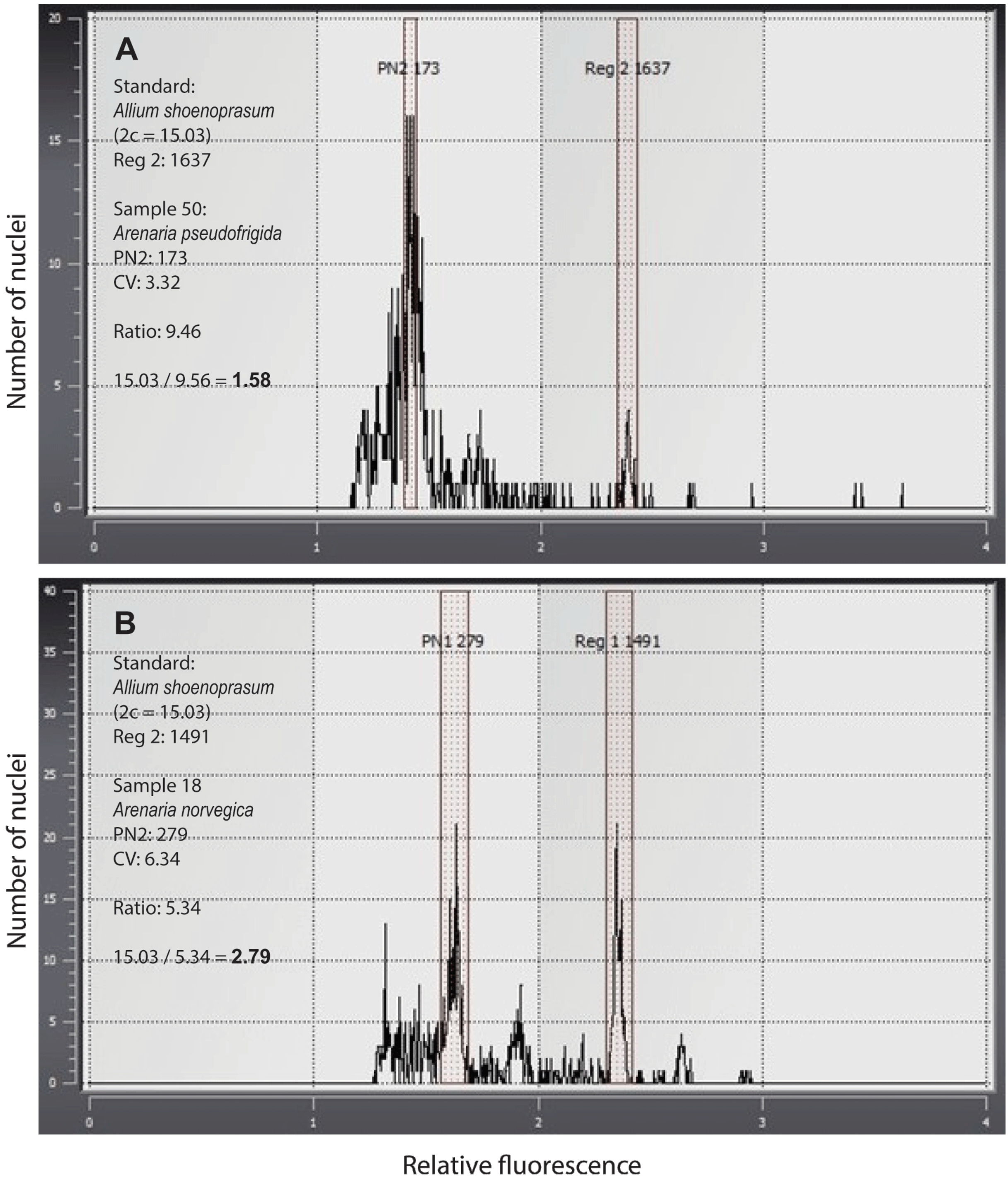

Figure S1. Examples of histograms of genome size estimations using flow cytometry. A: *Arenaria pseudofrigida* from Traill Island, Greenland. B: *Arenaria norvegica* from Latrabjarg, Iceland (Plant Cytometry Services, Didam, The Netherlands, www.plantcytometry.nl).
